# Ultrasound-stimulated microbubble enhancement of radiation treatments: endothelial cell function and mechanism

**DOI:** 10.18632/oncoscience.277

**Published:** 2015-12-15

**Authors:** Azza A. Al-Mahrouki, Emily Wong, Gregory J. Czarnota

**Affiliations:** ^1^ Department of Radiation Oncology, and Physical Sciences, Sunnybrook Health Sciences Centre and Sunnybrook Research Institute, Toronto, Ontario, Canada; ^2^ Departments of Radiation Oncology and Medical Biophysics, University of Toronto, Toronto, Ontario, Canada

**Keywords:** endothelial cells, bubbles, radiation, ultrasound, ceramide

## Abstract

Endothelial cell death caused by novel microbubble-enhanced ultrasound cancer therapy leads to secondary tumour cell death. In order to characterize and optimize these treatments, the molecular mechanisms resulting from the interaction with endothelial cells were investigated here.

Endothelial cells (HUVEC) were treated with ultrasound-stimulated microbubbles (US/MB), radiation (XRT), or a combination of US/MB+XRT. Effects on cells were evaluated at 0, 3, 6, and 24 hours after treatment. Experiments took place in the presence of modulators of sphingolipid-based signalling including ceramide, fumonisin B1, monensin, and sphingosine-1-phosphate. Experimental outcomes were evaluated using histology, TUNEL, clonogenic survival methods, immuno-fluorescence, electron microscopy, and endothelial cell blood-vessel-like tube forming assays.

Fewer cells survived after treatment using US/MB+XRT compared to either the control or XRT. The functional ability to form tubes was only reduced in the US/ MB+XRT condition in the control, the ceramide, and the sphingosine-1-phosphate treated groups. The combined treatment had no effect on tube forming ability in either the fumonisin B1 or in the monensin exposed groups, since both interfere with ceramide production at different cellular sites.

In summary, experimental results supported the role of ceramide signalling as a key element in cell death initiation with treatments using US/MB+XRT to target endothelial cells.

## INTRODUCTION

Conventional cancer therapies include: immunotherapy, chemotherapy, surgery, radiation, hormonal therapy, or a combination of these treatments. Despite the availability of many treatment approaches to treat diverse cancer types, a definite cure has not yet been reached. This is mainly due to the complicated biology of cancer, where hurdles such as treatment resistance and redundant cell signalling pathways are among contributors to treatment resistance.

For example, radiation therapy is widely used in treating many cancers, but because of radiation resistance, many research approaches aim to optimize different approaches to sensitize cancer cells to radiotherapy.

Recently, microbubbles have been used in a variety of cancer-treatment applications. Investigating the effect of cavitating microbubbles in concentrating energy and manipulating the cell membrane started more than a decade ago, and has improved the targeted delivery of drugs and genes, resulting in improved therapeutic applications [1]. Recent preclinical studies have investigated this phenomenon in cancer therapy and have demonstrated a high efficacy when treating colon cancer models [2].

Current reports indicate a significant sensitization of tumour cells when radiation is combined with ultrasound-stimulated microbubble exposure [3, 4, 5, 6, 7]. *In vitro* experiments using endothelial cells have also confirmed an enhancement of radiation effect [8] and activation of ceramide-related cell death, supporting the idea that these vascular cells are the primary target in this therapy. Evidence to date points to an important role of ceramide in such responses having demonstrated its production in response to ultrasound-stimulated microbubbles and radiation from experiments conducted *in vitro* and *in vivo*. Ultrasound-stimulated microbubble treatments have resulted in ceramide production in an exposure-dependent manner related to either increasing radiation doses or increasing exposure to ultrasound-stimulated microbubbles. Ceramide production has also been demonstrated to be directly linked to the development of tissue damage subsequent to vascular disruption caused by ultrasound-stimulated microbubbles [3, 4, 5, 6, 7]. Furthermore, cellular damage has been demonstrated to be prevented *in vitro* and *in vivo* by use of sphingosine-1-phosphate to counteract any ceramide-dependent induction of cell death. In order to be able to translate this potentially promising preclinical work to clinical use, a more in depth understanding of mechanisms involved is needed. In this study, cell death signalling pathways that involve ceramide are investigated.

Ceramide production and its involvement in signalling for cell death are associated with commonly used cancer therapies including chemotherapy and radiation therapy [9]. This includes its production either at the cellular membrane, as a ceramide enriched membrane domain [10, 11], or through the activation of ceramide *de novo* synthases [12]. Resistance to radiation therapy has been reported with sphingosine-1-phosphate (S1P) treatment, a potent signal-transduction molecule that induces cell survival, and is a metabolite of ceramide [11]. Further, therapy resistance has been also observed with fumonisin B1 exposure (an inhibitor of ceramide *de novo* synthesis) in the treatment of leukemic cells subjected to chemotherapy [12]. Additionally, a reported resistance of glioma cells to chemotherapy has indicated a rapid conversion of ceramide to glucosylceramide [13]. At a whole organism level, a defect in the acid sphingomyelinase gene can lead to Niemann-Pick disease, and lymphoblasts from patients with this disease have demonstrated resistance to ionizing radiation. Such resistance is believed to involve modulated ceramide production [14]. The treatment of actively dividing endothelial cells with ionizing radiation also results in ceramide-dependent apoptosis. Ceramide related cell death can also be modulated further; cells are protected when S1P is used, causing pro-survival signalling [15, 16]. When low concentrations of ceramide analogs have been used with HUVEC cells with lengthy exposure, both cell migration and proliferation have been inhibited [17]. All of this evidence suggests that ceramide signalling is important when investigating ceramide-dependent effects of ionizing radiation modality.

The work here represents an in-depth investigation of ultrasound-stimulated microbubbles treatments used in combination with radiation. This study demonstrates the effects these treatments have on endothelial cell structure and function, and investigates the involvement of ceramide-dependent signalling pathways. Specifically, the use of endothelial cells cultured to be resistant to radiation indicated that ceramide modulation is involved in radiation resistance.

## RESULTS

### Morphological changes with treatment

**Figure 1:**
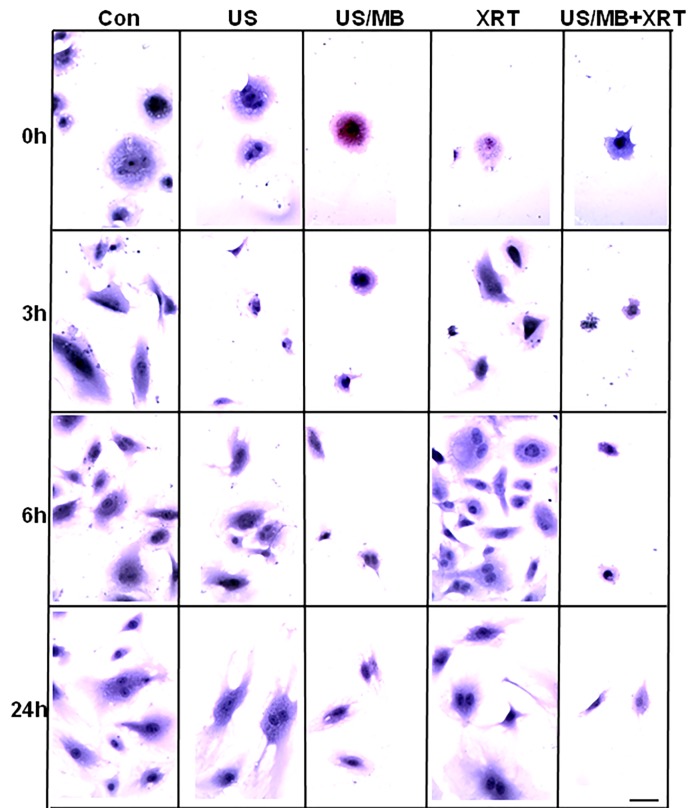
Light microscopy images of haematoxylin and eosin stained HUVEC cells Stained cells demonstrate treatment effect over 24 h time period, where both combined and single treatment of US/MB showed slow recovery compared to the other conditions. Magnification bar represents 50μm.

**Figure 2:**
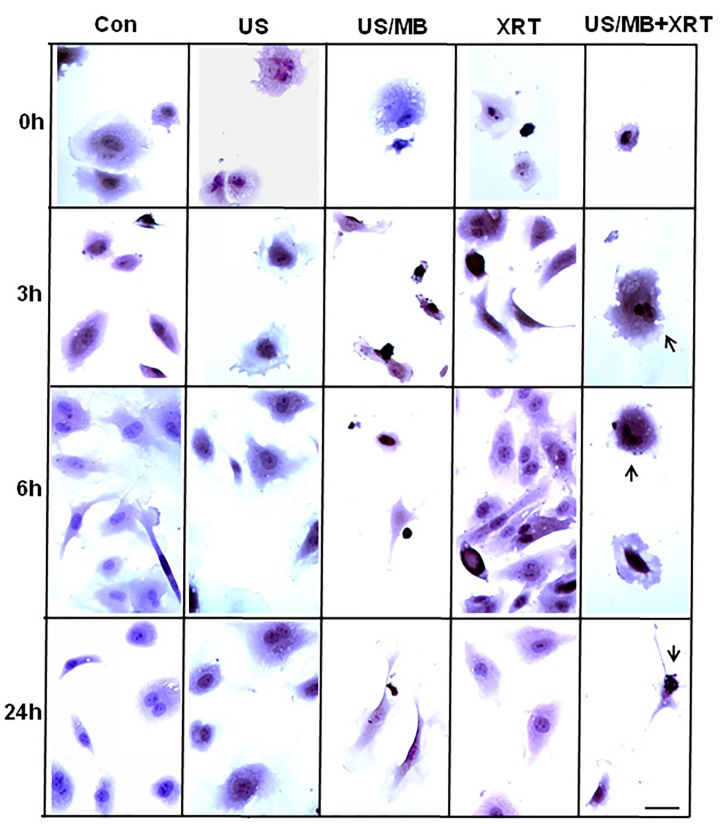
Light microscopy images of TUNEL-labelled HUVEC cells Labelled cells demonstrate treatment effect over 24 h time period, where both combined and single treatment of US/MB showed more fragmented DNA (brown label) compared to the other conditions. Magnification bar represents 50μm.

Cells exposed to treatment exhibited damage immediately after treatment (Figure 1). In particular, cells treated with ultrasound-stimulated microbubbles (US/MB) demonstrated nuclear pyknosis and membrane effects by 3 hours after treatment. Similarly, the combined treatment of MB and radiotherapy resulted in changes in cells which were visible in haematoxylin and eosin stained samples. Cells also underwent pyknotic changes shortly after radiation, consistent with a cell death appearance with remaining surviving cells frequently bi-nucleated twenty four hours after radiation exposure. Effects due to US/MB, XRT, and US/MB+XRT treatments were more visible in TUNEL staining for assessments of cell death (Figure 2). Control experiments with exposure to ultrasound alone (US) had no major discernable effect on cell appearance. Cells which were exposed to the combined treatment of ultrasound-stimulated microbubbles and radiation demonstrated different morphological results with more prominent formation of apoptotic bodies and positive TUNEL staining (Figure 1 and Figure 2). In particular, the labelling of the damaged DNA was persistent beyond six hours after treatments in both the US/MB and the US/MB+XRT conditions, but was not as evident in either the control or the US or the XRT treated cells (Figure 2).

**Figure 3:**
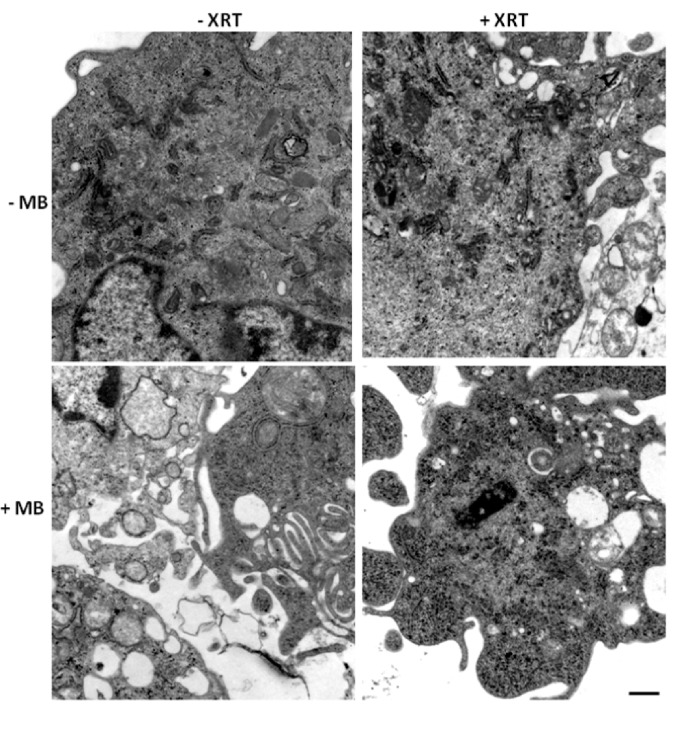
Electron micrographs of the control group under different treatment conditions Ultrastructure images illustrate vacuole formation and ribosomal clustering in irradiated cells, membrane disruption in US/MB treated cells, and late stage apoptotic condensed cells after treatment with US/MB+XRT. Magnification bar represents 500 nm.

Higher magnification ultrastructure studies were carried out to better visualize cellular morphology. Electron microscopy of treated cells also revealed more overt cellular damage with the combined treatment in comparison to treatment with single modalities alone. Treatment with ultrasound-stimulated microbubbles alone caused cell membrane distortion and the development of vacuolization inside cells (Figure 3). Similar damage was apparent with the combined treatments but not when radiation treatment alone was administered (Figure 3). Treatment with radiation alone or when administered with ultrasound-stimulated microbubbles also caused aggregations of ribosomes, which served as a cell death signalling marker (Figure 3).

**Figure 4:**
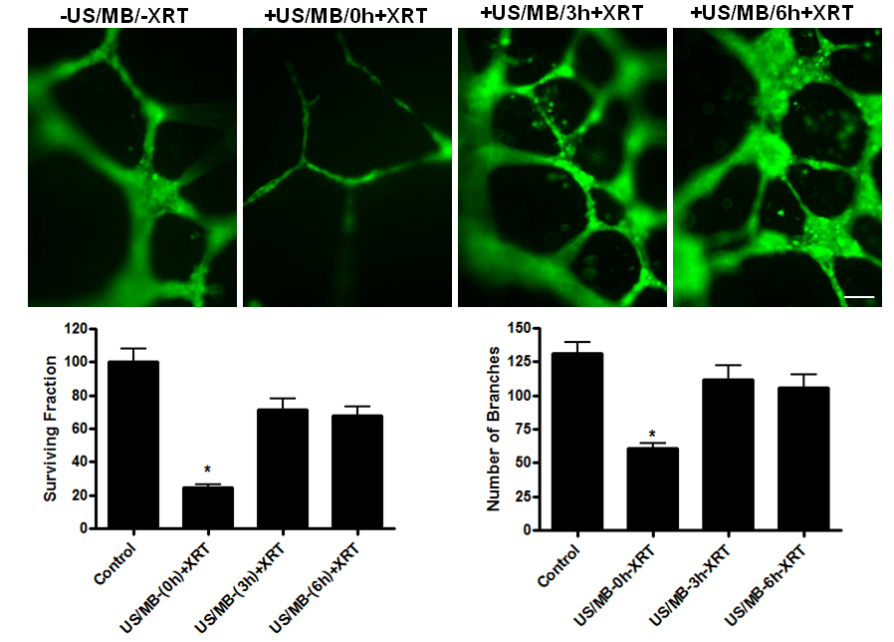
Time interval between the individual treatments of US/MB and XRT Both tube forming and clonogenic assays indicated maximum effects at zero hour interval, with less surviving cells and less formed branches. Magnification bar represents100μm.

In order to investigate the potential effect of timing on treatments at a cellular level, experiments were conducted with intervals introduced between the treatments. This was tested using functional assays that included not only clonogenic survival assays but endothelial-cell tube forming assays. Cells in suspension were treated with ultrasound-stimulated microbubbles followed by radiation at intervals of 0, 3, or 6 hours after the initial treatment. Significant cell death was only observed at the zero hour interval (P<0.0002). Furthermore, endothelial tube forming assay results also exhibited significantly less numbers of formed branches compared to the control at 0h (P<0.0001, Figure 4), but not at 3 or 6 hours indicating a time-dependent recovery *in vitro* which can begin to take place from the time of microbubble-stimulated treatments.

### Molecular mechanism determination

**Figure 5:**
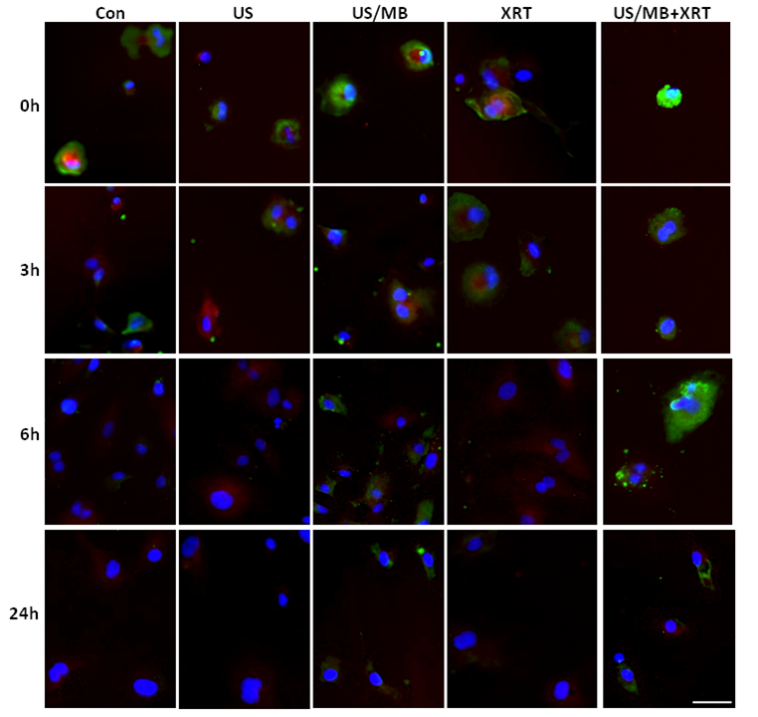
Changes in ceramide production over time Fluorescent labeling of ceramide (green) indicated an initial rise in levels of ceramide, which was attenuated over time as cells recovered. However, its production was persistent in both the US/MB and the combined treatments only. Labeling of mitochondrial complex IV (red) was dominant in recovering cells and some co-localization with ceramide was observed in both the US/MB and the combined treatments. Magnification bar represents 50μm.

Treatments were monitored for the production of ceramide as before (Figure 5) [8]. Exposure of cells to ultrasound-stimulated microbubbles caused an immediate increase in ceramide detected using immunohistochemistry. This appeared to diminish shortly after but was prominent again in immunohistochemistry, 6 hours later. This effect appeared persistent when radiation was used in combination with ultrasound-stimulated microbubbles with much more prominent immunostaining. Staining was stable for up to 24h afterwards with some apparent co-localization, with mitochondrial complex IV also observed, which indicates a mitochondrial apoptotic signalling.

Ceramide in cell stress can be generated from acid sphingomyelinase activity on the cell membrane but also through the *de novo* synthesis of ceramide. These two pathways were probed here using exogenously added chemical compounds which can interfere specifically in each of these pathways. Fumonisin-B1 was used as a blocker of ceramide *de novo* synthesis. Monensin was used as a blocker of ceramide biosynthesis by inhibiting acid sphingomyelinase activity and ceramide Golgi-based transport. Endothelial cells were grown in the presence of 10 mM glutamine which rendered them relatively radio-resistant such that an 8Gy dose resulted in only 20% cell death and 80% survival, different when compared to results in the absence of glutamine [8].

**Figure 6:**
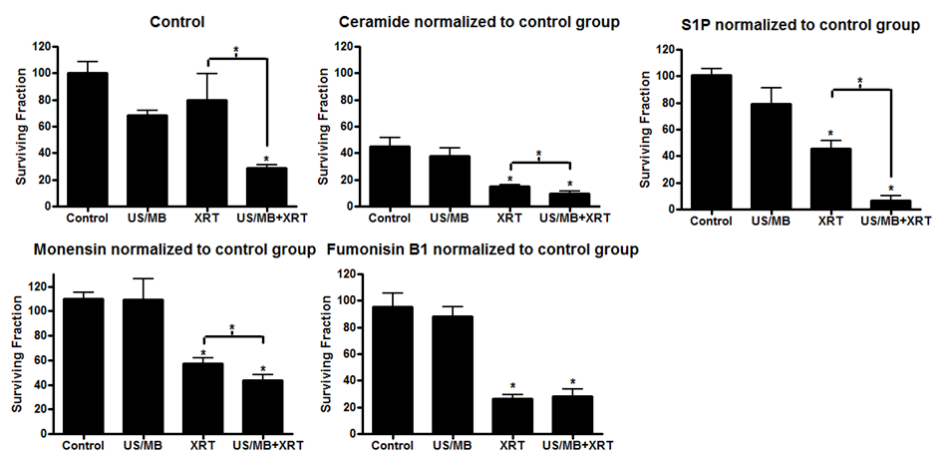
Clonogenic survival assays and ceramide modulation The use of exogenous ceramide attenuated survival levels when compared to the control group. Compared to the modulation by ceramide, enhanced survival was observed when using S1P (enhances cellular survival), monensin, or fumonisin B1 (both monensin and fumonisin B1 inhibit ceramide production at different sites). However, decreased levels of survival were observed with the combined treatment under all of the modulators.

**Figure 7:**
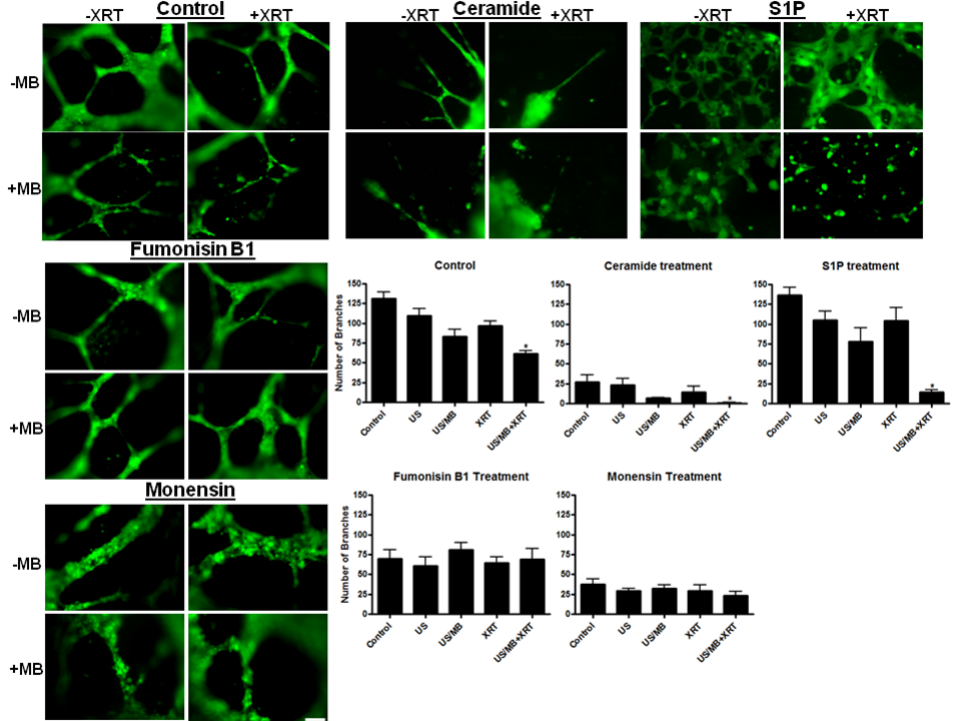
Images and summary of formed tubes The control group showed well formed tubes and loops with the non-treated and the XRT conditions, but some disruption was observed under the US/MB and combined treatments. The ceramide treated group showed general failure of tube formation, with few to no formed loops, especially under the combined treatment. The S1P treated group showed increase of tube formation, with more formed branches and small loops. However, a disruption of the formed tubes was observed with combined treatments. The fumonisin B1 treated group illustrated large well defined formed tubes with large loops. This observation was consistent under the different treatment conditions including the combined treatment of US/MB+XRT. The monensin treated group illustrated large formed tubes with large loops, but generally less than what was observed in the case of fumonisin B1 treated group. This observation was consistent under the different treatment conditions, including the combined treatment of US/MB+XRT. Magnification bar represents 100μm. The analyses of these images indicated significantly lowered levels of formed tubes in the control, ceramide, and S1P groups, but not with monensin or fumonisin-B1 treatments when combined with US/MB+XRT.

**Figure 8:**
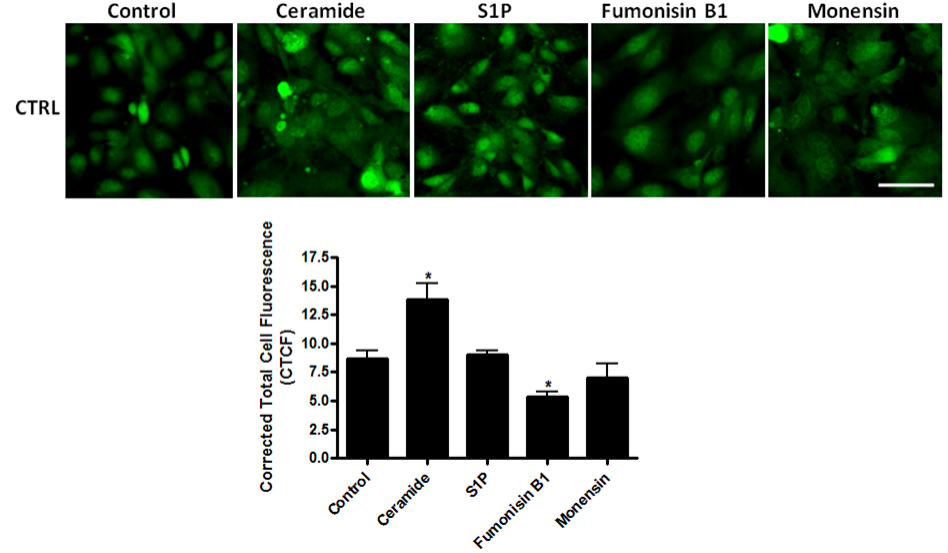
Fluorescent labeling of ceramide in control cells that were not treated or were treated with different modulators Levels of ceramide were elevated in the ceramide treated cells and were attenuated in the S1P, monensin, or fumonisin B1 treated cells when compared to the control cells. Magnification bar represents 50μm.

**Figure 9:**
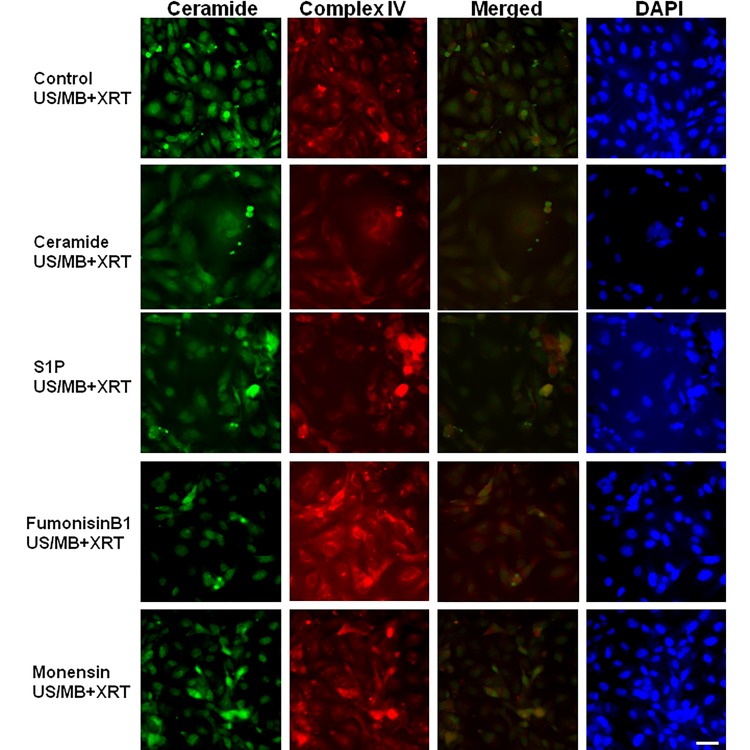
Fluorescent triple labeling of ceramide, complexIV, and DAPI in US/MB+XRT treated cells Levels of ceramide were elevated in the ceramide treated cells and were attenuated in the S1P, monensin, or fumonisin B1 treated cells when compared to the control cells. Complex IV labeling was strongest in cells treated with fumonisin B1, indicating abundant presence of mitochondria. Magnification bar represents 50μm.

**Figure 10:**
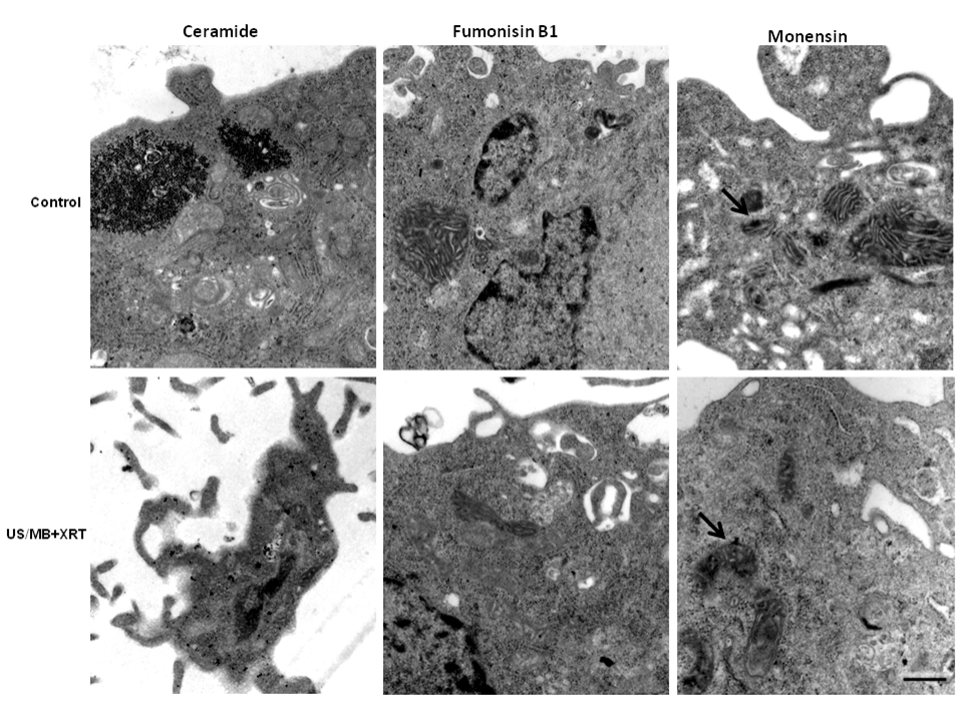
Ultrastructure images of cells treated with ceramide or fumonisin B1, or monensin Cells treated with ceramide alone show ribosomal clustering, a feature signalling for cell death, combing ceramide exposure with the bubble treatment led to late stage apoptosis. Fumonisin B1 treated cells show treatment resistance when exposed to the combined treatment of US/MB+XRT. Monensin treated cells show condensed electron particles in their mitochondria (arrows) in both the monensin treated control and the monensin combined with US/MB+XRT. Magnification bar represents 500 nm.

Endothelial cells were exposed to various components, which effect ceramide-related cell signalling to investigate their role on cell responses to membrane-based cell perturbations. Specifically, exogenous ceramide (C2), sphingosine-1-phosphate (a ceramide metabolite), and monensin (inhibitor of acid sphingomyelinase response) and fumonisin B1 were used (inhibitor of *de novo* ceramide synthesis). Outcomes were assessed using clonogenic survival assays, tube forming assays, immuno-fluorescence, and electron microscopy (Figures 6–10).

In the group of experimental samples not exposed to any exogenous agent, the combined condition of US/ MB+XRT demonstrated a significant decrease in cellular survival with *P*< 0.0002 when compared to its control or with *P*< 0.0043 when compared to XRT alone. Survival in the XRT sample was higher as cells were cultured in the presence of 10 mM glutamine compared to earlier work without glutamine [18]. The use of exogenous ceramide led to further cell death in all conditions when compared to the conditions of the control group with more than a 50% decline in cell survival (Figure 6). A similar decrease under the combined condition was also observed in the groups treated with monensin, or fumonisin B1 with *P*< 0.05, however, the relative proportionate responses were different between conditions. Treatment with monensin or fumonisin B1 resulted in the samples exposed to ultrasound-stimulated microbubbles (US/MB) having similar survivals compared to control samples appearing to having cell death inhibited. Exposure to these two agents also diminished the differences between samples exposed to radiation alone (XRT) and the combined treatment (US/MB+XRT) from a ratio of 5:2 for untreated samples, to 5:4 and 5:6 for samples exposed to monensin and fumonisin B1, respectively. Data on S1P has been published elsewhere [5, 18] and primarily inhibits cellular damage promoting cell survival overall (Figure 7). As before, S1P exposure rescued endothelial cells from US/MB effects. Although survival was reduced with treatment with XRT alone and with the combined treatments, the cellular ability to form tubes was maintained with the XRT condition with S1P exposure. Previous rescue to US/MB with respect to survival was apparent, but with a 2 Gy radiation dose. Here, no rescue of tube-forming ability took place with US/MB+8Gy treatment.

Tube forming assays were used in this study to further validate the effect of the different modulators on endothelial cell function. In experiments with samples not exposed to any exogenous agent, impairment of tube formation was observed in the combined treatments, in the samples not exposed to any chemical agents (*P*<0.0001), in ceramide-exposed (*P*<0.0031), and in S1P-exposed (*P*<0.0286) samples (Figure 7). Exposure to ceramide led to depressed tube formation in all treatment groups. Exposure to S1P resulted in improved tube formation in conditions relative to control with the exception of the combined treatment (US/MB+XRT) where it lowered tube forming ability. Exposure to fumonisin-B1 (inhibitor of de novo ceramide formation) resulted in larger well defined tubes with large lumen when compared to control samples, and because the regions of interest (ROIs) were kept constant through the analysis, number of branches were generally reduced, however, the functional ability to form well defined tubes were maintained even under the combined condition. Similarly, exposure to monensin (inhibitor of acid sphingomyelinase activity) resulted in tube formation which was well defined, also with larger lumen relative to the fumonisin-exposed samples.

**Figure 11:**
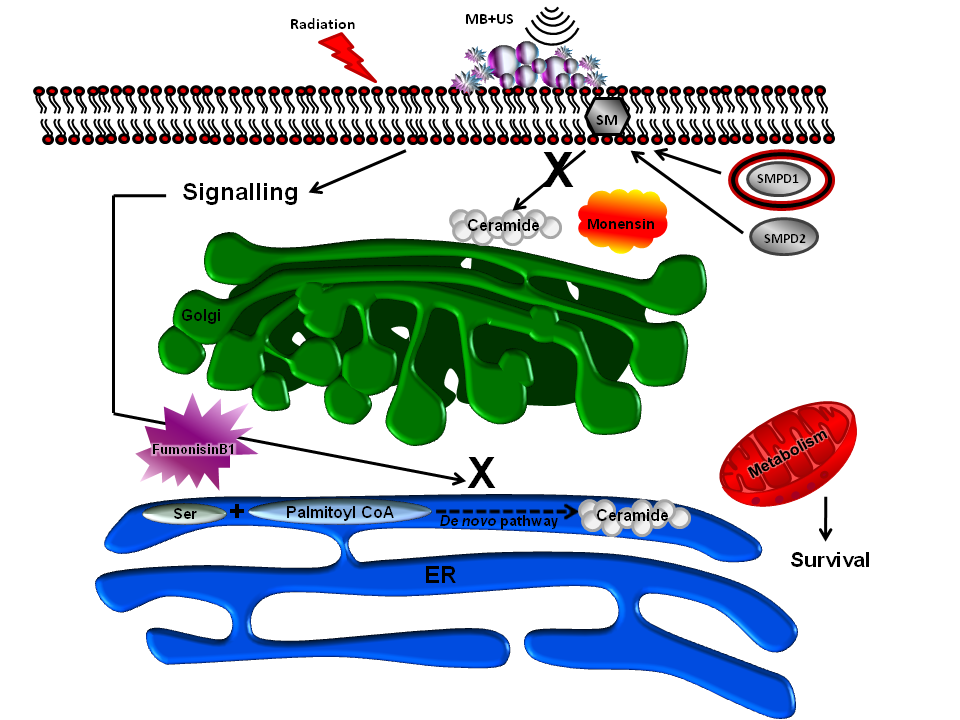
Schematic summary of the effect of ceramide modulators The use of ceramide modulators (either monensin or fumonisin B1) to attenuate ceramide production, leads to enhanced endothelial cell function/survival, when treated with US/MB, XRT or with both.

Ceramide immuno-fluorescence staining was carried out to validate changes in ceramide levels as a result of using the different modulators (Figure 8). This, as expected, indicated an increase in ceramide in the ceramide treated group, and a decrease in both the fumonisin-B1 and the monensin treated groups (Figure 8). In Figure 9, staining for mitochondria and nuclear position (DAPI) is presented. This indicated, in part, co-localization between ceramide and mitochondria, observed in the combined treatment (US/MB+XRT) in the different chemical agent-exposed groups, suggesting the involvement of the mitochondria in the response to ultrasound-stimulated microbubble exposure. This was depressed, as expected, in the fumonisin treated sample. In order to qualitatively investigate this further, electron microscopy was used where late stage apoptosis was observed in the combined treatment samples (Figure 10). This also revealed ribosomal aggregation in the cytoplasm of ceramide treated cells, a cell death signal. On average, cells appeared less damaged with fumonisin B1 exposure than with monensin treatment. Electron dense particles were observed in the mitochondria of cells subjected to monensin (Figure 10). Monensin and fumonisin B1 treatment resulted in less observable damage than with US/MB+XRT treatment carried out without either of these chemical agents (Figure 3). A schematic summary demonstrating the effects of ceramide modulators is presented in Figure 11.

## DISCUSSION

The use of ultrasound-stimulated microbubble therapy to enhance radiation effect in tumours is a new approach that can potentially prove to be very valuable as a cancer treatment modality. It may permit the use of lower radiation doses for the same effect as higher radiation doses, in terms of tumour control. When using *in vivo* animal-based tumour models, ultrasound-stimulated microbubble treatments combined with radiation have been demonstrated to cause 40-60% tumour cell death within 24 hours [5]; a magnitude of radiation enhancement typically not seen with chemical radiation enhancing or sensitizing agents. Experiments have also indicated superior animal survival in therapy regimens with multiple-fraction treatments for the use of ultrasound-stimulated microbubbles used in conjunction with radiation [5].

An understanding of the biological mechanism of action at a cellular level of this radiation enhancement is very important in its optimization. Endothelial cells lining tumour blood vessels are believed to be the primary target in such treatments [5, 19]. In that paradigm, in terms of mechanism, endothelial cell death subsequently leads to tumour cell death. This model is being recognized recently as an important target of radiation treatment [10, 20]. Tumour vascular content has also been recognized as important in radiation treatments [21], and other aspects of radiation biology such as radiation resistance in glioblastoma multiforme have been reported to be caused in part by resistance of tumour microvascular endothelial cells [22].

In this present work, HUVEC, an experimental model system for endothelial cells supplemented with glutamine, a known radiation protector, was used to further investigate microbubble therapy effects and to characterize pathways affected by ultrasound-stimulated microbubbles used in conjunction with radiation therapy. The working hypothesis for this type of therapy [3, 5, 8] is that stimulation of microbubbles with ultrasound leads to the disruption of endothelial cell membranes, leading to the activation of sphingomyelinases to produce ceramide. The subsequent action of ceramide on the mitochondria leads to the initiation of apoptotic signalling and endothelial cell death, and this potential mechanism is investigated here. Supplementation with glutamine and steroid in culture was to permit the use of a large radiation dose 8 Gy yet to be able to have sufficient cell survival.

Effects of combining the different therapy components (US/MB, XRT) *in vitro* appeared to be additive as before [5, 8]. A recovery of cells from any stress or damage imparted to them from treatments appeared to start 6 hours after treatments and peaked at 24 hours later. An exception to this was microbubble treatments in the absence of or with radiation (US/MB and US/MB+XRT). Furthermore, cell survival and tube formation were significantly reduced with the combined treatment. This is in agreement with previous reports on the efficacy of this therapy on endothelial cells and subsequently, tumour cells [2, 3, 5, 8]. Effects i*n vivo* were synergistic [5] in comparison to *in vitro* results due to secondary ischemic effects of endothelial cell death and vascular collapse which do not take place *in vitro*.

Combined treatments could be more effective in overcoming resistance due to the involvement of diverse alternative cell death signalling pathways. Therefore, if a given apoptotic signalling pathway has a defect that contributes to resistance, another can be potentially be activated [23].

Maximizing the effect of the combined treatments here is essential. This was investigated through varying time intervals between the ultrasound-stimulated microbubble therapy and radiation treatments. Previous results *in vivo* indicated an optimal time of 6 hours between the ultrasound and radiation-based treatments, but it remained to be investigated whether this was a direct endothelial effect, or secondary due to collapse of tumour vasculature.

Results in this study demonstrate that the maximum cell death and the lowest level of tube forming activity were achieved at zero hour intervals. This indicates at a single cell level that a time delay does not further enhance effects as it does *in vivo* for other reasons. For other microbubble-based treatments, similarly, no time interval was reported to be most effective when combing gene transfer therapy and ionized radiation in prostate cancer treatments [24], which is in agreement with the findings here. Both radiation treatments and ultrasound-stimulated microbubble treatments resulted in the production of ceramide, potentially explaining the additive effect *in vitro*. *In vivo,* where this type of cell effect leads to vascular collapse and secondary tumour cell ischemia [5], it is expected that there will be a different time-based effect.

Treatment with high dose ionizing radiation is known to involve increased apoptotic-ceramide signalling in endothelial cells [16]. Similarly, elevation of apoptotic-ceramide signalling has been reported with microbubble-based therapy, and is thought to be caused by the effect of the ultrasound-stimulated microbubbles on the membranes of endothelial cells [3, 5, 6, 8]. Ceramide is a lipid signalling molecule that mediates many cellular functions including migration, differentiation, induced apoptosis, senescence, and autophagy [25]. It is either produced *de novo*, or from the metabolism of different molecules at the cellular membrane, or elsewhere in cells, where many enzymes are involved in its synthesis [26]. This raises the question of whether the ultrasound-stimulated microbubble-based apoptotic effect is a result of signalling through ceramide production at the cellular membrane, which then targets the mitochondria to initiate the apoptotic signal, or, if microbubble-based stress on the membrane leads to the stimulation of *de novo* ceramide production, which could possibly signal through mitochondrial or non-mitochondrial pathways.

In order to investigate this, a number of modulators were used here and combined with various treatment conditions. Modulators included exogenous ceramide and S1P. These two modulators exerted contrasting effects, with ceramide leading to more cell death and S1P enhancing survival, which is in agreement with previous reports [5, 8, 27]. With exception of the combined ultrasound-stimulated microbubble and radiation treatment samples (US/MB+8Gy); cells treated with S1P were not rescued. This could possibly be either because cell death signalling overrode any cell recovery, or as previously noted, S1P protects dividing endothelial cells only from ceramide-induced apoptosis, but not from DNA-damage-related mitotic death that the use of combined ultrasound-stimulated ultrasound and radiation modalities may have caused [15]. Additional modulators investigated included monensin, which inhibits acid sphingomyelinase activity lowering ceramide production at the membranes by blocking ceramide transport through the Golgi, and fumonisin B1, which inhibits the *de novo* synthesis of ceramide. Our results indicate that both modulators protected the functional ability of cells to form tubes, albeit slightly differently, even when exposed to the combined treatment (US/MB+XRT). Furthermore, this was despite a significant level of cell death as observed from the clonogenic assays used to assess cell survival. Nevertheless, mitochondrial damage associated with the release of high levels of dense electron particles that was more evident in monensin treated cells but not fumonisin-B1 treated cells. In other research, along the same lines, oligodendrocytes had a reduction in apoptotic cell death when subjected to ionizing radiation when treated with monensin, but not when treated with fumonisin B1 [28]. In contrast, fumonisin-B1 suppressed cell death signalling in bortezomib (proteasome inhibitor) treated pancreatic cancer cells [29]. This indicates differential ceramide signalling pathways with irradiation or chemical agents when studied in different cell models. As for experiments in this study, both *de novo* and membrane-produced ceramide appeared to contribute to cell death in treated endothelial cells. Further, apoptotic cell death signalling here seems to involve mitochondrial damage because of the observed reduced labelling of the mitochondrial complex IV with exogenous ceramide exposure, and increased labelling with either fumonisin-B1 or monensin exposure. Ceramide mitochondrial cell death signalling was reported to be through the permeabilization of the mitochondrial outer membrane and the formation of ceramide barrel-stave channels across the membrane, where pro-apoptotic proteins interact with ceramide to increase membrane permeability [30]. This facilitates the release of cytochrome c and the initiation of apoptotic signalling.

## CONCLUSIONS

In conclusion, this study is the first in depth analysis of the signal transduction pathways involved in signalling for cell death with ultrasound-stimulated microbubble therapy, alone or in combination with ionizing radiation. This therapy was initially developed to increase the sensitivity of cancer cells to radiation therapy in a focused, targeted approach to reduce radiation dose, creating a more effective therapy with minimal side effects. Ceramide formation through acid sphingomyelinase-based cell signalling and *de novo* synthesis were both involved in the effects of ultrasound-stimulated microbubbles when combined with radiation as a treatment. The understanding of this therapy and the characterization of the involved cell death signalling pathways *in vitro* should help in its development, optimization, and ultimate use as a treatment for cancer patients.

## MATERIALS AND METHODS

### Cell cultures

Human umbilical vein endothelial cells (HUVEC) were obtained from American Type Culture Collection, ATCC (PCS-100-010; Manassas, VA, USA). Cells were grown in the presence of 10 mM glutamine as a supplement in the growth media to render them more resistant to radiation effects in order to better modulate ceramide related cell signalling. Under these conditions an 8 Gy dose of radiation results in 50-60% cell survival (an order of magnitude of protection compared to an 8 Gy dose in the absence of glutamine). Cells were cultured in flasks pre-coated with 0.1% gelatin and grown using endothelial cell growth kit-VEGF medium (PCS-100-041 from ATCC).

Cells were grown and maintained under humidity at 37°C, 5% CO2, and were used for experiments between passages three and six. Confluent cells were harvested using 0.05% Trypsin-EDTA from Invitrogen (25300-062, Carlsbad, CA) or trypsin-EDTA for primary cells (PCS-999-003) and trypsin neutralizing solution (PCS-999-004), both obtained from ATCC (Manassas, VA, USA). Cells were then collected by centrifugation at 4°C for 7 min (180 × G/1000 rpm) and were counted using a hemocytometer. To ensure reproducibility, all experiments were carried out in triplicate.

### Ultrasound stimulated microbubbles and radiation treatments

Experiments were carried out as before [8]. Approximately 3.0 × 10^6^ cells in 1.5 ml of medium were subjected to ultrasound-stimulated microbubble and radiation exposure. Definity microbubbles (Lantheus Medical Imaging, MA, USA) were activated by shaking at 3000 rpm for 45 seconds using a Lantheus Vial-shaker device. The treatment set up consisted of an acquisition system (Acquiris CC103), an amplifier (RPR4000, Ritec), a wave-form generator (AWG520, Tektronix) and an ultrasound transducer (Valpey Fisher Inc., MA, USA) with 500 kHz central frequency. It was focused at 8.5 cm, and at the focal point the -6 dB beam width was 31 mm.

Samples were exposed to ultrasound of 16-cycles tone burst with 3 kHz pulse repetition frequency for 30 seconds, with a 10% duty cycle to avoid any heating. Cells were constantly stirred at 240 rpm to allow adequate exposure to the ultrasound waves. For microbubble-ultrasound treatments, 3.3% (v/v) Definity microbubbles (6×10^8^ microbubbles) were added and exposed to 500 kHz ultrasound with 570 kPa peak negative pressure corresponded to a mechanical index of 0.8. Combined treatments were carried out by ultrasound exposure first and then irradiation of samples within two minutes. Cells were irradiated using 160kV (Faxitron X-ray CP-160 cabinet X-radiator system, Faxitron X-ray Corporation, IL, USA) to deliver 8 Gy at a rate of 200 cGy/minute. Cells were used for immunohistochemistry, clonogenic survival assays, functional assays, and for structural assessments as described below.

Longitudinal experimentation was carried out in order to assess the outcome of the effect of the different treatment conditions on cells. Results were analyzed at 0, 3, 6, and 24 hours after treatments, to enable the monitoring of cellular recovery under different treatment conditions. To optimize the time interval when using the combined treatments, cells were first exposed to bubble therapy, and then, at time intervals of 0, 3, and 6 hours, were followed by exposure to radiation. This was to investigate the most effective outcome *in vitro* using the combined therapy of bubbles and radiation.

### Molecular mechanism determination

In order to investigate signalling for cell death through ceramide activation, a number of modulators were used in experimental conditions including: C2-ceramide (to amplify the effect of ceramide), sphingosine-1-phosphate (to block ceramide mediated apoptosis), fumonisin-B1 (to block de novo synthesis of ceramide), and monensin (to inhibit acid sphingomyelinase signalling, which is involved in ceramide production). All modulators were used at a final concentration of 1μM, and cells were incubated at 37<deg>C for 2-3 hours. Cells were then harvested and treated with combinations of radiation and ultrasound-stimulated microbubbles as described above. All chemicals were obtained from Sigma Aldrich (St. Louise, MO). Several approaches were used to evaluate the experimental outcomes including: histology, TUNEL, immuno-fluorescence, electron microscopy, clonogenic survival assays, and tube forming functional assays as described below.

### Clonogenic assays

After subjecting cells to different treatment conditions, 1.0 × 10^3^ cells were plated in 35 mm dishes in triplicate and incubated at 37°C and 5% CO2 for up to five days to develop colonies. The formed colonies were fixed and stained with 0.3% methylene blue/methanol (v/v) for 20 minutes at room temperature. The numbers of the counted colonies were compared and Mann-Whitney test was used to determine the statistical significance.

### Endothelial tube forming assay (*in vitro* angiogenesis)

Control and treated cells (3 × 10^4^) were added onto endothelial cell matrix (ECM) gel obtained from Cell Biolabs (San Diego, CA, USA). The assay was carried out according to the manufacturer's instructions. Cells from each condition were added in triplicate in a 96-well sterile plate, and were incubated at 37<deg>C for about 18 hours. The growth medium was gently removed, and formed tubular structures were fluorescently labelled with Calcein AM. A Zeiss Axiovert 200 M inverted fluorescent microscope and AxioVision software 4.6 were used to examine the formed tubes. Obtained images were processed and analyzed using Angiogenesis Analyzer, an ImageJ application (http://rsb.info.nih.gov/nih-imageJ; National Institutes of Health, Bethesda, MD, USA). Averaged numbers of branches from at least three images per well from three wells per condition were computed. The resulting numbers from different conditions were compared and a Mann Whitney test was used to determine the statistical significance.

### Histology, immunohistochemistry and microscopy

Treated or control cells (1.0 × 10^4^) were plated on glass cover slips pre-coated with 0.1% gelatin, and placed in 12-well cell culture. Plates were then incubated at 37°C and 5% CO2 for two to three hours. After incubation, media was removed and cells were fixed in freshly prepared 1% (v/v) paraformaldehyde for twenty minutes at room temperature, which was followed by several washes with PBS and then samples were stained with haematoxylin and eosin. Samples were also labelled with TUNEL staining for cell death, or labelled using immunohistochemistry for ceramide and mitochondrial complex IV. Staining using TUNEL was carried out using an apoptosis detection kit (GenScript, Piscataway, NJ, USA), and the labelling was done according to the manufacturer's instructions. For ceramide and complex IV labeling, cells were washed with PBS and permeabilized with 0.1% triton in PBS. Antigen retrieval was done by incubating cells in cytonin for 10 min. Non-specific background was blocked with 10% non-immune goat serum, and was followed by incubation with the primary antibodies for 1 h at room temperature. After several washes with PBS/0.1% Tween20, incubation with cy3, anti-mouse and cy5, anti-rabbit secondary antibodies was carried out for 30 min at room temperature. DAPI was used as a counterstain. The primary antibody to ceramide (ALX-804-196) was obtained from Alexis (San Diego, CA, USA) and that to complex IV (AP51039PU-N) was obtained from Acris antibodies (San Diego, CA, USA). Both antibodies were used at 1:20 dilution. The fluorescently labeled secondary antibodies were obtained from GE Healthcare Life Sciences (Baie d'Urfe, Quebec, Canada) and were used at 1:50 dilution.

For all light or fluorescent microscopy stained cells were imaged using either a LEICA DM LB light microscope and Leica IM1000 software, or with a fluorescent Zeiss Axiovert 200 M inverted microscope and AxioVision software, version 4.6.

### Electron microscopy

After treatments, cell samples were also collected by centrifugation and fixed in formaldehyde/glutaraldehyde, 2.5%/2.5% (v/v) in PBS. This was followed by fixation with 1% osmium tetroxide (w/v). Samples were subsequently dehydrated and embedded using Embed-812 kit, according to the manufacturer's instructions. After the polymerization of the resin, thick (0.6μm) and thin (70nm) ultra-sections were prepared using an ultramicrotome (Reichert-Jung). Thick sections were mounted to a glass slide and stained with 1% (v/v) toluidine blue for quick evaluation. Thin sections were collected on copper grids and stained the following day using 2% uranyl acetate and 0.1% lead citrate. Samples were allowed to dry and images were obtained using a JEOL 1011 transmission electron microscope operating at 80 kV and at 25,000X magnification. All materials and chemicals were obtained from Electron Microscopy Sciences (PA, USA).
